# Characterization of cardiac- and respiratory-driven cerebrospinal fluid motion based on asynchronous phase-contrast magnetic resonance imaging in volunteers

**DOI:** 10.1186/s12987-017-0074-1

**Published:** 2017-09-27

**Authors:** Ken Takizawa, Mitsunori Matsumae, Saeko Sunohara, Satoshi Yatsushiro, Kagayaki Kuroda

**Affiliations:** 1 Department of Neurosurgery, Tokai University School of Medicine, 143 Shimokasuya, Isehara, Kanagawa 2591193 Japan; 2 Course of Science and Technology, Graduate School of Science and Technology, Tokai University, 4-1-1 Kitakaname, Hiratsuka, Kanagawa 2591292 Japan

**Keywords:** Cerebrospinal fluid, Magnetic resonance imaging, Fluid dynamics, Phase-contrast image, Quantitative analysis

## Abstract

**Background:**

A classification of cardiac- and respiratory-driven components of cerebrospinal fluid (CSF) motion has been demonstrated using echo planar imaging and time-spatial labeling inversion pulse techniques of magnetic resonance imaging (MRI). However, quantitative characterization of the two motion components has not been performed to date. Thus, in this study, the velocities and displacements of the waveforms of the two motions were quantitatively evaluated based on an asynchronous two-dimensional (2D) phase-contrast (PC) method followed by frequency component analysis.

**Methods:**

The effects of respiration and cardiac pulsation on CSF motion were investigated in 7 healthy subjects under guided respiration using asynchronous 2D-PC 3-T MRI. The respiratory and cardiac components in the foramen magnum and aqueduct were separated, and their respective fractions of velocity and amount of displacement were compared.

**Results:**

For velocity in the Sylvian aqueduct and foramen magnum, the fraction attributable to the cardiac component was significantly greater than that of the respiratory component throughout the respiratory cycle. As for displacement, the fraction of the respiratory component was significantly greater than that of the cardiac component in the aqueduct regardless of the respiratory cycle and in the foramen magnum in the 6- and 10-s respiratory cycles. There was no significant difference between the fractions in the 16-s respiratory cycle in the foramen magnum.

**Conclusions:**

To separate cardiac- and respiratory-driven CSF motions, asynchronous 2D-PC MRI was performed under respiratory guidance. For velocity, the cardiac component was greater than the respiratory component. In contrast, for the amount of displacement, the respiratory component was greater.

## Background

Intracranial cerebrospinal fluid (CSF) motion changes with cardiac and respiratory rhythms [1]. In clinical practice, most clinicians accept that the motion of the CSF has two elements, a fast movement synchronized with the heartbeat and a somewhat slower movement synchronized with respiratory movements, on the basis of observations of the fluid surface during surgery or CSF drainage. When discussing the physiological role of CSF, analyzing its motion in terms of its separate cardiac and respiratory components is valuable for elucidating the pathologies of diseases that cause abnormal movement of the CSF, such as hydrocephalus. Magnetic resonance imaging (MRI) provides a noninvasive technique for studying CSF dynamics in human subjects [2–6]. Numerous researchers have investigated cardiac modulation of CSF using various MRI techniques [2, 6, 7]. On the other hand, only a few studies of the modulation of CSF motion induced by respiration have been performed [8–10]. To visualize the cardiac- and respiratory-driven CSF motions separately, Yamada et al. [8] used a spin-labeling technique called time-spatial labeling inversion pulse (Time-SLIP). Chen used the simultaneous multi-slice (SMS) echo planar imaging (EPI) technique [11] based on MRI. A new approach using frequency analysis has recently also come into use. Yatsushiro et al. [12] used the 2-dimensional phase-contrast (2D-PC) technique to classify intracranial CSF motion into cardiac and respiratory components and expressed these by means of correlation mapping.

We consider that quantitative analysis of velocity and displacement, the integral of velocity over time, is required to ascertain the dynamics of CSF motion as water, and this study was conceived on the assumption that quantitative analysis of CSF motion by 2D-PC, a development building on previous techniques, is appropriate for this purpose. To separate the cardiac and respiratory components of CSF motion, the asynchronous real-time 2D-PC technique was used in seven healthy volunteers under controlled respiration. The velocity and the amount of displacement of the cardiac and respiratory components of CSF motion were quantified. The velocity and displacement were then compared in each respiratory cycle, and the effects of respiratory and cardiac components on CSF motion were quantitatively investigated.

## Methods

Our institutional review board approved this research. All volunteers were examined after providing appropriate informed consent, consistent with the terms of approval from the institutional review board of our institution.

Asynchronous 2D-PC technique under controlled respiration was performed in 7 healthy volunteers (6 male and 1 female) aged 21–31 years. The respiratory cycle was set to 6, 10, and 16 s, to cover the range of the normal respiratory cycle. Volunteers were requested to control their respiration according to audio guidance for inhalation and exhalation timing. To monitor respiration, a bellows-type pressure sensor was placed around the abdomen of the subject, and an electrocardiogram (ECG) was monitored to identify the frequency distribution of individual cardiac motion. Asynchronous 2D-PC steady-state-free precession (SSFP) was performed on a 3-T MR scanner with the following conditions: flow encode direction foot–head (FH); data points 256; repetition time (TR) 6.0 ms; echo time (TE) 3.9 ms; flip angle (FA) 10°; field of view (FOV) 28 × 28 cm^2^; velocity encoding (VENC) 10 cm/s; acquisition matrix 89 × 128 (half-Fourier); reconstruction matrix 256 × 256; and slice thickness 7 mm. These conditions yielded a frame rate of 4.6 images/s (temporal resolution of 217 ms). The total duration of data acquisition for each subject was 55 s. After obtaining the color-coded velocity vector images, rough outlines of the ROI were specified around the Sylvian aqueduct and the foramen of Monro. The partial volume effect arising from the relatively large voxel size (approximately 2 mm) used in the present experiment made a simple threshold-based segmentation of the T_2_-weighted image difficult. To segment the CSF regions on the images with a reduced partial volume effect and to apply these images to the velocity and pressure images as masks for the quantitative analyses, a novel segmentation technique, called spatial-based fuzzy clustering, was applied. The details of this technique are explained elsewhere [13].

The waveform in the individual voxels was separated into respiratory and cardiac components based on frequency range, and the maximum velocity was determined for the respective components. The technical details of the procedure were explained in our previous study [12, 14].

The ratio of the individual velocity of the respiratory or cardiac component to the sum of the velocities of the respiratory and cardiac components was calculated for both velocity and displacement. The results of the above calculations for the cerebral aqueduct and the foramen magnum were compared statistically. Equation 1 shows the formula for the calculation of the fraction, *F*_r_, of the velocity of the respiratory component to the sum of the velocities for the respiratory and cardiac components.

1 $$F_{\text{r}} = \frac{{v_{\text{r}} }}{{v_{\text{r}} + v_{\text{c}} }}$$

where *v*_r_ is the respiratory component of the velocity, while *v*_c_ is the cardiac component.

The mean CSF displacement of each component in the cranial and caudal directions was calculated from the velocity waveform based on the following equation,

2 $$D = \frac{1}{N}\sum\limits_{n = 1}^{N} {\left( {\Delta t\sum\limits_{m = 1}^{M} {v\left( {m \cdot \Delta t} \right)} } \right)}$$

where *v*(*m*∙ Δ*t*) is the velocity at the mth time point of the observation with a sampling period of Δ*t*, and *M* is the number of time points in the cranial or caudal direction. For example, when the velocity was positive, its direction was regarded as cranial, and the number of corresponding data points was set to *M*. *N* is the number of voxels in a region of interest (ROI) for the displacement measurement. Fractions of cardiac- and respiratory-induced displacements were calculated in a similar manner with equation [1], but separately for the cranial and caudal directions.

The Kolmogorov–Smirnov test and the Mann–Whitney *U* test were used to compare the respiratory and cardiac components of the velocity and the amount of displacement.

## Results

**Fig. 1:**
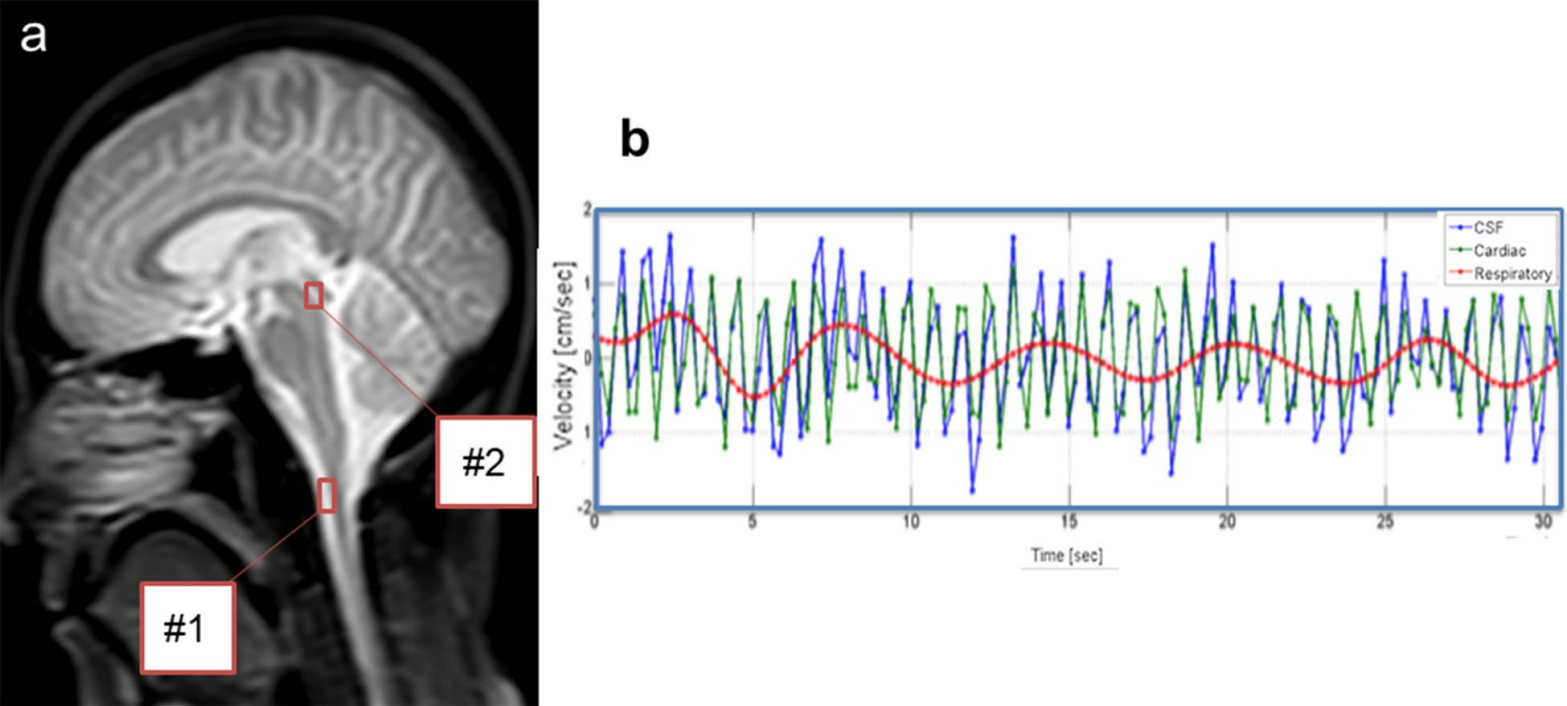
A *T*_2_-weighted image (**a**) of a healthy subject with 2 ROIs (red rectangles) placed in the foramen magnum (#1) and the Sylvian aqueduct (#2). The temporal changes of the total velocity wave of the CSF, and separated the cardiac and respiratory velocity components at ROI #1 are shown in (**b**)

**Fig. 2:**
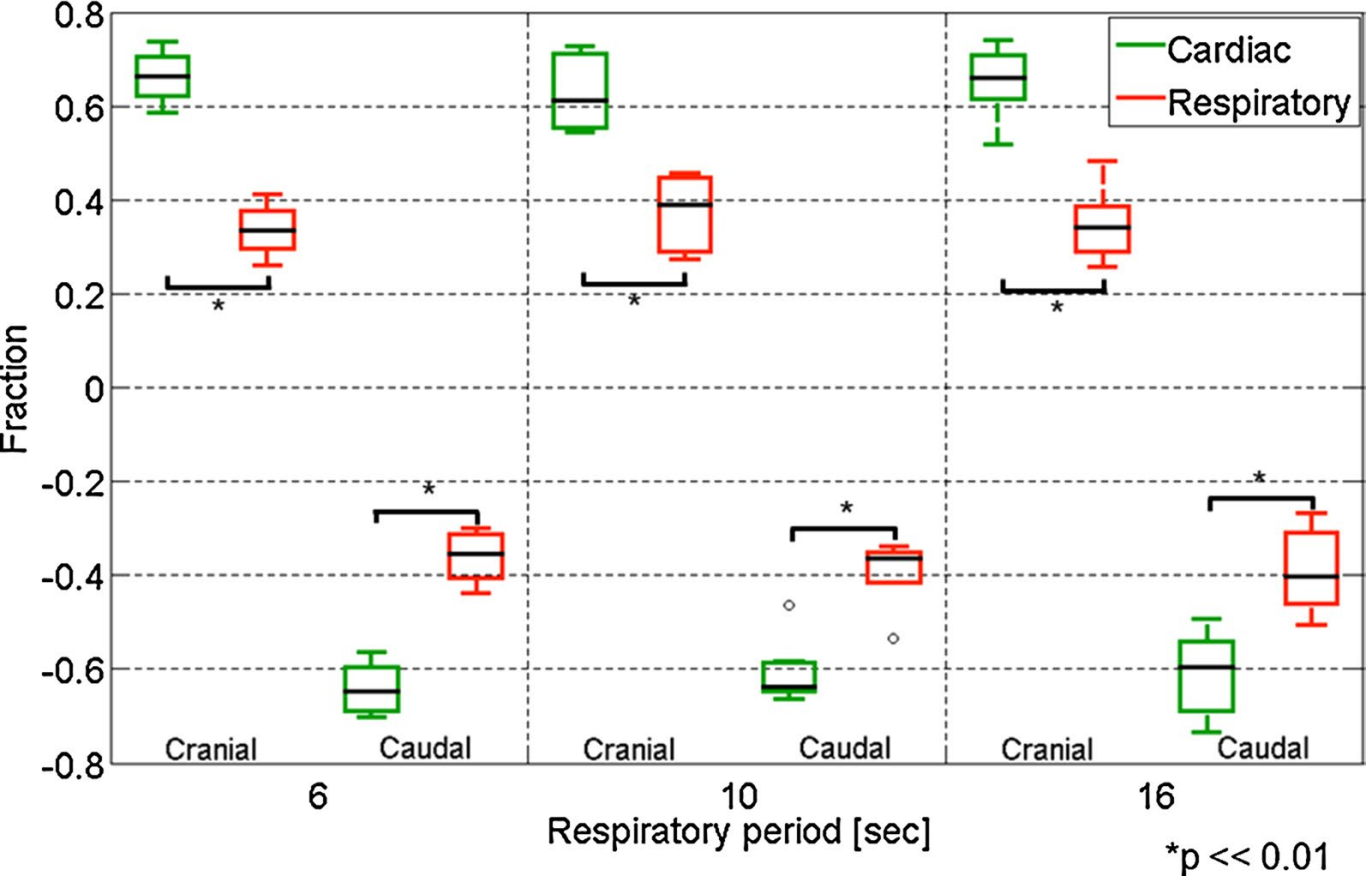
Box plots of the fractions of the respiratory and cardiac components of the CSF velocity in the three different respiratory cycles (6, 10, and 16 s) at the aqueduct. The cranial and caudal directions are plotted separately. Outlying values are indicated by “o”

**Fig. 3:**
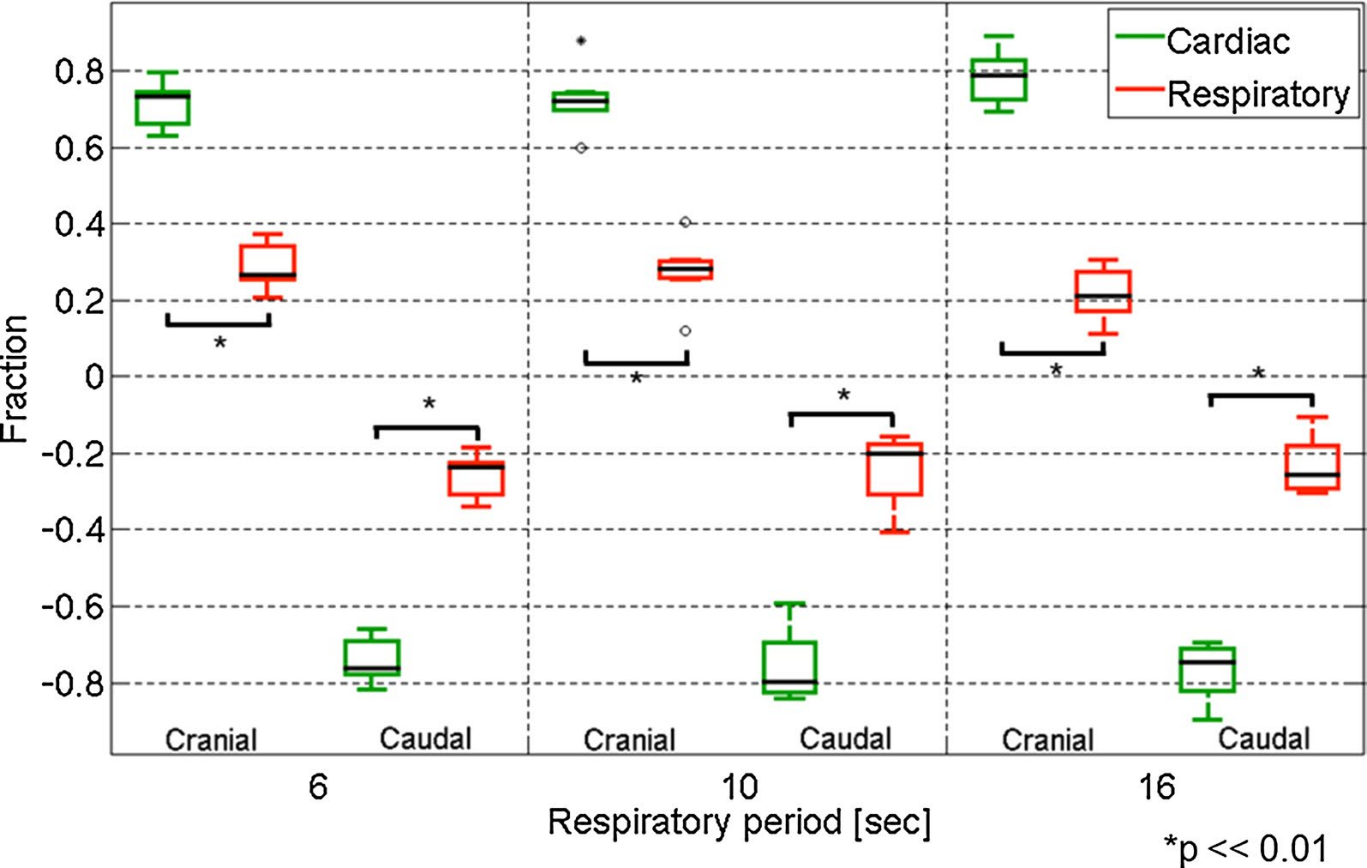
Similar box plots of the fractions of the CSF velocity components as Fig. 2 but at the foramen magnum. Outlying values are indicated by “o”, and far-outlying values are indicated by an asterisk

Figure 1b presents a CSF velocity waveform obtained with a 6-s respiratory cycle by the asynchronous time-resolved 2D-PC technique at region of interest (ROI) #1 placed at the foramen magnum, as depicted in Fig. 1a. Summary of the velocities and displacement of the respiratory and cardiac components of the CSF at the Sylvian aqueduct and the foramen magnum are shown in Tables 1, 2. The fractions of the respiratory and cardiac components of the CSF velocity at the Sylvian aqueduct are shown in Fig. 2. The cardiac component was significantly greater than the respiratory component (*p* = 0.002) regardless of the respiratory period. A similar plot for the fractions at the foramen magnum is shown in Fig. 3. In results for both the Sylvian aqueduct and the foramen magnum, the cardiac component was significantly greater than the respiratory component (*p* = 0.002) throughout the three different respiratory cycles. There was no significant difference between the fractions of the different respiratory periods for both the respiratory and cardiac components.

**Table 1:** Summary of the cardiac- and respiratory-driven CSF velocities (cm/s) in the cranial and caudal directions for the three different respiratory periods

Respiratory period (s)	Cranial		Caudal	
	Cardiac	Respiratory	Cardiac	Respiratory
Sylvian aqueduct				
6	0.216 ± 0.049	0.109 ± 0.025	−0.219 ± 0.058	−0.121 ± 0.020
10	0.209 ± 0.061	0.119 ± 0.016	−0.212 ± 0.052	−0.137 ± 0.028
16	0.201 ± 0.041	0.110 ± 0.031	−0.205 ± 0.045	−0.131 ± 0.033
Foramen magnum				
6	0.948 ± 0.431	0.384 ± 0.194	−0.976 ± 0.466	−0.348 ± 0.232
10	1.003 ± 0.534	0.359 ± 0.178	−1.028 ± 0.511	−0.301 ± 0.095
16	1.008 ± 0.540	0.246 ± 0.095	−1.008 ± 0.489	−0.275 ± 0.117

Velocity (cm/s) in the aqueduct and foramen magnum

Values are shown as mean ± standard deviation

**Table 2:** Summary of the cardiac- and respiratory-driven CSF displacements (cm) in the cranial and caudal directions for the three different respiratory periods

Respiratory period (s)	Cranial		Caudal	
	Cardiac	Respiratory	Cardiac	Respiratory
Sylvian aqueduct				
6	0.051 ± 0.022	0.124 ± 0.023	−0.049 ± 0.019	−0.121 ± 0.024
10	0.054 ± 0.027	0.138 ± 0.043	−0.052 ± 0.023	−0.140 ± 0.052
16	0.054 ± 0.025	0.147 ± 0.054	−0.053 ± 0.024	−0.156 ± 0.058
Foramen magnum				
6	0.319 ± 0.154	0.505 ± 0.314	−0.313 ± 0.147	−0.489 ± 0.325
10	0.334 ± 0.169	0.614 ± 0.355	−0.331 ± 0.169	−0.670 ± 0.362
16	0.308 ± 0.133	0.501 ± 0.281	−0.308 ± 0.132	−0.572 ± 0.424

Displacement (cm) in the aqueduct and foramen magnum

Values are shown as mean ± standard deviation

**Fig. 4:**
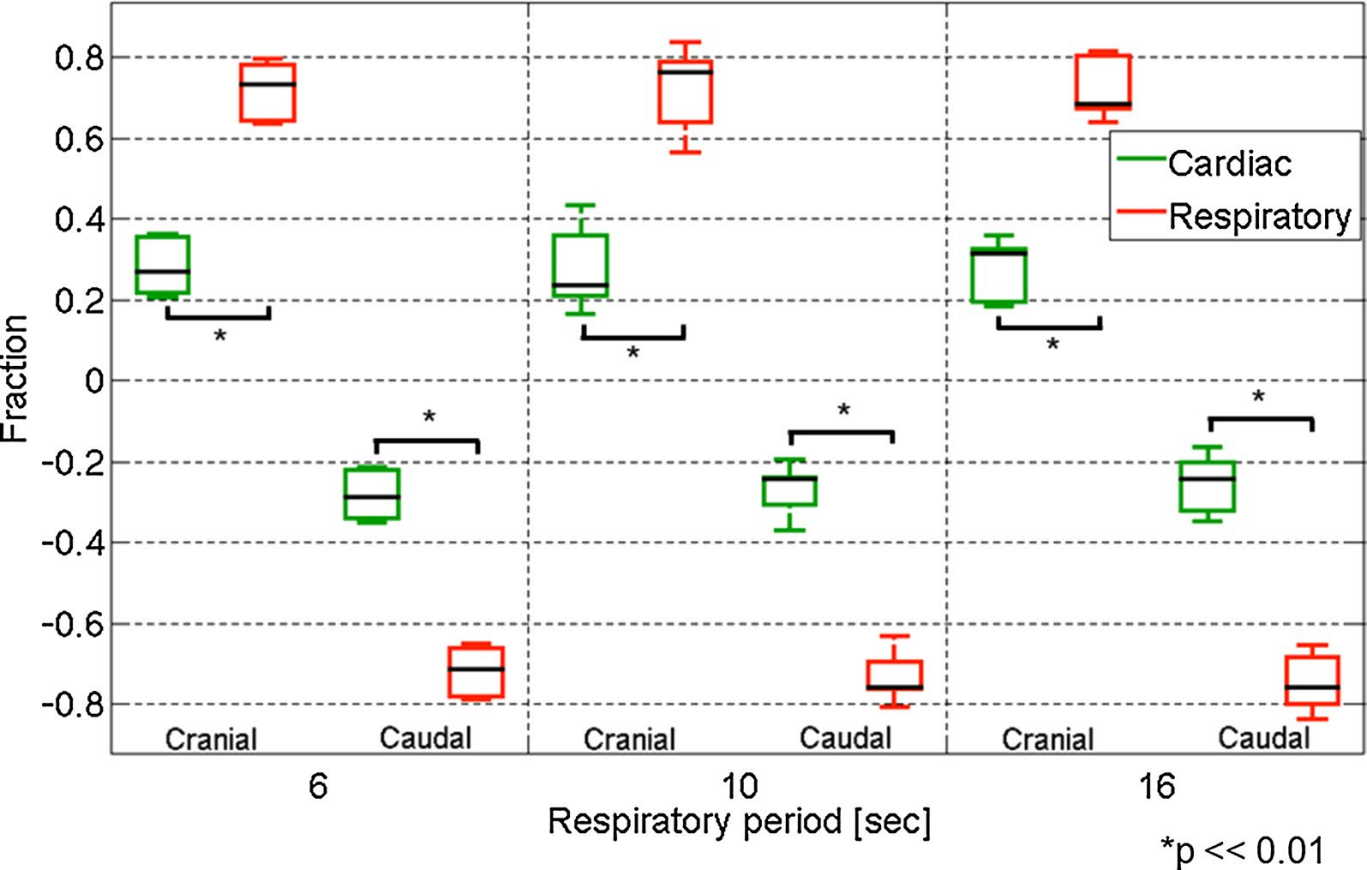
Box plots of the fractions of the respiratory component and the cardiac component of the cranial and caudal displacements at the aqueduct. The cranial and caudal directions are plotted separately

**Fig. 5:**
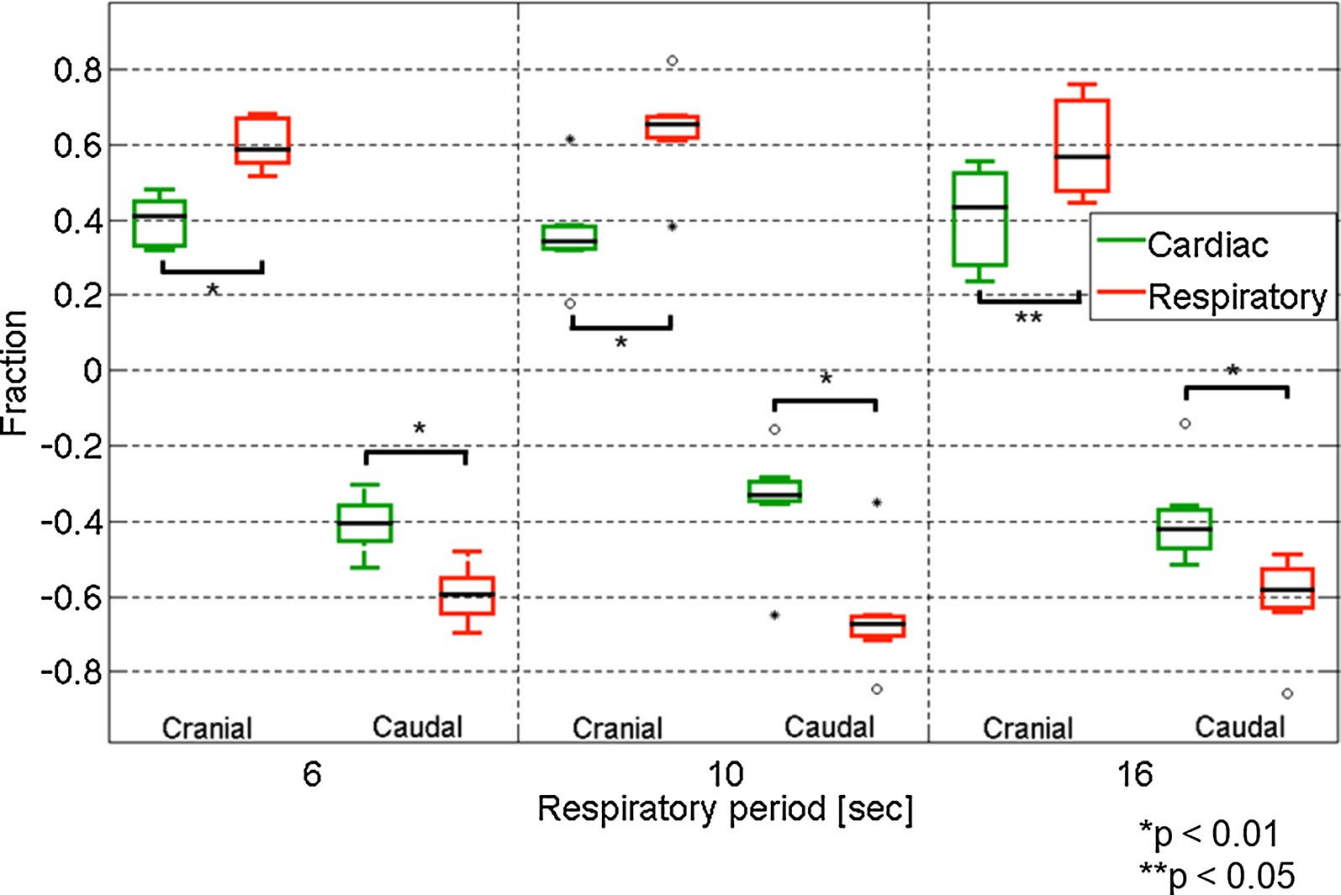
Similar box plots as Fig. 4 for the displacement fractions at the foramen magnum. Outlying values are indicated by “o”, and far-outlying values are indicated by an asterisk

The fraction of the displacement of the CSF for the respiratory and cardiac components at the Sylvian aqueduct is shown in Fig. 4. Throughout the respiratory cycle, the respiratory component was significantly greater than the cardiac component (*p* = 0.002). No significant difference was found between the fractions of the different respiratory periods. A similar plot for the displacement fraction at the foramen magnum is shown in Fig. 5. In this region, the displacement fraction of the respiratory component was significantly greater than that of the cardiac component in the respiratory cycle at 6 and 10 s (*p* = 0.02). However, no significant difference was observed at 16 s (*p* = 0.85). Significant differences between the respiratory cycles of 6 and 16 s were observed in both the respiratory and cardiac components (*p* = 0.004). No differences were observed in the other respiratory cycles.

## Discussion

To understand the driving force of CSF motion, researchers have investigated animals and humans using a variety of techniques [1]. Many concluded that CSF pulsations are mainly arterial in origin. On the other hand, CSF flow changes due to respiration have been the subject of only a few MRI studies. However, some researchers have investigated the effects of respiratory motion on CSF flow using MRI techniques [8, 10, 11, 15]. Beckett et al. [15] used simultaneous multi-slice (SMS) velocity imaging to investigate spinal and brain CSF motion. They reported that the CSF motion in the spine and brain is modulated not only by cardiac motion, but also by respiratory motion. Chen et al. [11] used SMS EPI technique under respiratory guidance to measure respiratory- and cardiac-modulated CSF velocity and direction. They concluded that, during the inspiratory phase, there is upward (inferior to superior) CSF movement into the cranial cavity and lateral ventricles, with a reversal of direction in the expiratory phase. Yamada et al. [8] investigated the effect of respiration on CSF movement by using a non-contrast Time-SLIP technique with balanced steady-state-free precession (bSSFP) readout. Their results demonstrated that a substantially greater amount of CSF movement occurs with deep respiration than with cardiac pulsations. Later, Dreha-Kulaczewski et al. [10] concluded that inspiration is the major regulator of CSF motion. Dreha-Kulaczewski et al. [10] used a highly under-sampled radial gradient–echo sequence with image reconstruction by regularized nonlinear inversion (NLINV) for observing the effect of respiratory on the CSF motion. Since signal intensity modulation due to the inflow effect was used in their work, separated and direct quantification for the CSF velocities due to the cardiac pulsation and respiration were not performed. In the recent publication, Yildiz et al. [9] used very similar technique with our present work to quantify and characterize the cardiac and respiratory-induced CSF motions at the level of the foramen magnum. Assessment of intracranial CSF motions was, however, not made in their work. Thus we believe our present work is adding new insights concerning on the cardiac and respiratory-induced CSF motions in the intracranial space. In the present study, we differentiated the cardiac and respiratory components to evaluate CSF motion. One of the simplest ways to separate cardiac and respiratory motions is to understand frequency analysis. Sunohara et al. [14] developed a method using 2D-PC to analyze the driving force of CSF in terms of power and frequency mapping and successfully analyzed the cardiac and respiratory components of CSF motion, albeit obtaining their images from volunteers engaged in controlled respiration. Our frequency technique was taken further for quantitative analysis of CSF motion related to cardiac and respiratory components. The mathematical algorithm for separating the cardiac and respiratory components of the CSF motion is described in our previous work [12]. Shortly, Fourier transformation was applied to the time series of the obtained velocity data at each voxel. The components of CSF motion were extracted from the frequency spectrum by selecting the particular frequency bands corresponding to the cardiac and respiratory frequencies. In this particular work, the frequency band for the cardiac component was set as 1.0–1.6 Hz, while that for the respiratorion was 0.018–0.3 Hz.

In the present study, CSF motion was separated into respiratory and cardiac components. The amount of CSF displacement was found to be larger in the respiratory component than in the cardiac component in both cranial and caudal directions. Simultaneously, while the cardiac component showed a smaller displacement, the velocity was higher compared to the respiratory component. In other words, the movement of CSF due to the cardiac component was rapid and small, and that due to the respiratory component was slow and large. These results are consistent with those of the visual analysis of CSF reported by Yamada et al. [8] demonstrating that the influence of the respiratory component on the amount of displacement per unit of time was greater than that of the cardiac component. These findings provide quantitative values for results that will be readily understandable to clinicians who have observed the rapid, short-period, powerful CSF motion synchronized with the heartbeat and the slowly pulsing, long-period CSF motion in clinical practice. The difference in the displacement was significant (p < 0.001) and clear in the Sylvian aqueduct for all respiratory periods. The difference became slightly less clear in the foramen magnum, particularly for longer respiratory periods (p < 0.05 for the 16-s cycle). This may be attributed to the fact that the respiratory process tended to be unstable in the longer period (16 s), and, thus, the individual variation among the volunteers became larger than that in the shorter period.

Time-SLIP enables changes in spin to be visualized. This approximates the results for displacement shown in the present study, showing that CSF moves long distances in accordance with respiratory variations. In the present results, the velocity indicated the rapid movement of CSF with a short period associated with the heartbeat. To summarize CSF motion on the basis of these results, although CSF moves fast as it spreads around the vessels with the heartbeat, it moves over comparatively long distances in accordance with the slower movements of breathing, and this fast movement and movement over long distances may be responsible for physical exchanges in the brain and spinal cord.

However, the physical quantity measured in the present study is the displacement calculated by integrating the CSF velocity in the cranial or caudal direction, unlike the spin traveling distance, which the spin-labeling technique measures. Another important point is that the temporal resolution for data sampling (217 ms/frame) was not high enough to sample the cardiac-driven motion. Assuming a heart rate of 1 Hz, only 4–5 points can cover a cycle of cardiac-driven CSF motion resulting in a lack of waveform sampling accuracy, although the present technique is a quantitative measurement based on the 2D-PC technique, which can measure the fluid velocity with 10% accuracy [16].

## Conclusions

In this study the asynchronous 2D-PC method was used under respiratory guidance, which also enabled the evaluation of the respiratory movement element. This was done by performing 2D-PC scanning continuously without a trigger in order to evaluate the slow, long-period motion of CSF and then carrying out quantitative analysis. The feature of the PC method in combining the time element with velocity and direction makes it possible to observe the complex motion of the CSF, providing the next step forward in elucidating the physiological functions of the CSF in vivo. The cardiac-related CSF motion is predominant over the respiratory-related motion, which maintains CSF pressure in the CSF cavity. However, the CSF moves a long distance, as shown by our analysis of displacement. The displacement of CSF in different cavities is important to exchange substances between the parenchyma and the CSF space. During surgery, neurosurgeons frequently see powerful short-range cardiac-related CSF waves and long range, large-wave rhythmical pulsations related to the ventilator. Furthermore, at the tip of external ventricular drainage, clinicians always see the short-range, short-distance CSF pulsation and the long-range, long-distance CSF pulsation, and this alternate CSF pulsation can be identified using the present technique non-invasively. Our final goal was to identify the pathogenesis of CSF circulatory disturbances, as in hydrocephalus and Alzheimer dementia. Using quantitative analysis, we were able to differentiate the subgroup of disease or do a pre- and post-treatment analysis. One of the limitations is that the present MR technique is vulnerable to changes in the position of the human body. Such a position change makes the CSF motion more complex, resulting in failure to assess the association between human movements and CSF motion in daily life.

## Abbreviations

CSF: cerebrospinal fluid
Time-SLIP: time-spatial labeling inversion pulse
MRI: magnetic resonance imaging
2D: 2-dimensional
PC: phase-contrast
2D-PC: 2-dimensional phase-contrast
EEG: electrocardiogram
SSFP: steady-state-free precession
FH: foot-head
TR: repetition time
TE: echo time
FA: flip angle
FOV: field of view
VENC: velocity encoding
ROI: region of interest
SMS: simultaneous multi-slice
EPI: echo planar imaging
bSSFP: balanced steady-state-free precession
